# Infection- and Vaccine-Induced SARS-CoV-2 Seroprevalence, Japan, 2023

**DOI:** 10.3201/eid3006.231454

**Published:** 2024-06

**Authors:** Ryo Kinoshita, Sho Miyamoto, Shoko Sakuraba, Jun Sugihara, Motoi Suzuki, Tadaki Suzuki, Daisuke Yoneoka

**Affiliations:** National Institute of Infectious Diseases, Tokyo, Japan (R. Kinoshita, S. Miyamoto, M. Suzuki, T. Suzuki, D. Yoneoka);; Ministry of Health, Labour, and Welfare, Tokyo (S. Sakuraba, J. Sugihara)

**Keywords:** COVID-19, SARS-CoV-2, viruses, respiratory infections, zoonoses, vaccine-preventable diseases, seroprevalence, surveillance, seroepidemiology, infant, children, elderly, maternal immunity, Japan

## Abstract

We assessed SARS-CoV-2 seroprevalence in Japan during July–August 2023, with a focus on 2 key age groups, 0–15 and >80 years. We estimated overall seroprevalence of 45.3% for nucleocapsid antibodies and 95.4% for spike antibodies and found notable maternally derived spike antibodies in infants 6–11 months of age (90.0%).

Before the Omicron variant emerged, <5% of Japan’s population had experienced SARS-CoV-2 infection (*1*), but ≈34 million (27%) COVID-19 cases were reported by May 7, 2023 (*2*). The mandatory reporting of all cases burdened the healthcare system, prompting Japan to adopt a sentinel surveillance approach starting May 8, 2023. Sentinel surveillance may restrict representativeness because of its limited coverage population and overlooks persons with mild or no symptoms because they often remain undiagnosed. Persons >6 months of age have been recommended to receive COVID-19 vaccines in Japan, where mRNA vaccines (Pfizer-BioNTech, https://www.pfizer.com; Moderna, https://www.modernatx.com) are predominantly used. Seroepidemiologic surveys can aid in understanding ascertainment rate and assessing the real-time prevalence of antibodies resulting from both natural infection and vaccination (*3*).

Since 2020, Japan’s Ministry of Health, Labour, and Welfare has conducted 6 rounds of seroepidemiologic surveys among residents (survey 1) (*1*,*4*) and 4 rounds among blood donors (survey 2) (*4*,*5*). Survey 1 analyzed both nucleocapsid (N) and spike (S) antibody seroprevalence through random samples of participating residents (>20 years of age), whereas survey 2 focused solely on N antibody seroprevalence among blood donors (16–69 years of age). Those surveys revealed that older and at-risk age groups exhibit lower infection rates and higher vaccination rates as well as a relatively lower seroprevalence before Omicron emergence compared with the United Kingdom and United States (*5*). However, the surveys excluded infants, children, and the elderly. To complement the previous surveys, our study aimed to assess SARS-CoV-2 seroprevalence among outpatients, focusing on 2 key age groups (*6*–*8*): the younger generation (0–15 years) and the older generation (>80 years).

## The Study

We used residual blood samples from local clinics collected across 22 prefectures in Japan during July 22–August 21, 2023, to investigate SARS-CoV-2 antibody seroprevalence across persons 0 to 101 years of age. This study, conducted in accordance with the Act on the Prevention of Infectious Diseases and Medical Care for Patients with Infectious Diseases (*9*), did not require a formal ethics review or participant consent.

From various private testing companies offering testing services for nonbed clinics across regions, 1 was chosen to closely mirror Japan’s population density and age distribution. We conducted random sampling from the collected pool of residual specimens among 11 age groups (0–4, 5–9, 10–14, 15–19, 20–29, 30–39, 40–49, 50–59, 60–69, 70–79, and >80 years) until 385 specimens were sampled per age group, assuming a 50% (+5%) seroprevalence, with a 5% α-error. We measured N antibodies by using Elecsys Anti-SARS-CoV-2 (Roche, https://www.roche.com) and S antibodies by using Elecsys Anti-SARS-CoV-2 S (Roche). We used a cutoff index of 1.0 U/mL to determine the presence of N antibodies and 0.8 U/mL to determine the presence S antibodies, as determined by the manufacturer. We assumed S antibody concentrations measured <0.4 U/mL to be 0.2 U/mL. We also collected data on age, sex, and prefecture. To adjust for each prefecture’s age and sex distribution, we used weighted tabulation on the basis of the October 2022 baseline population (*10*). We computed 95% CIs by using the binomial exact method.

**Figure 1:**
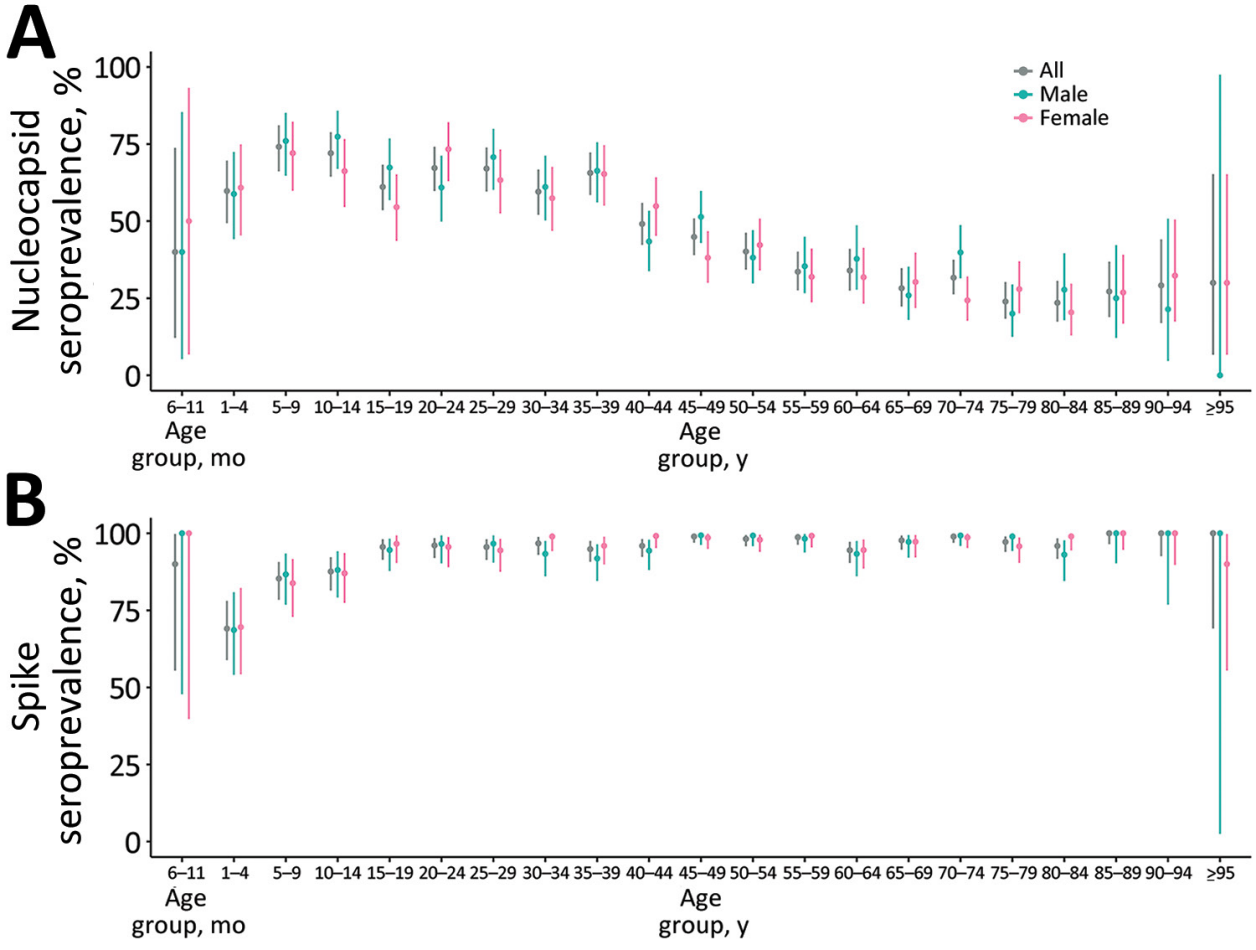
Age-stratified weighted seroprevalence of nucleocapsid and spike SARS-Cov-2 antibodies in Japan, 2023. A) Weighted seroprevalence of nucleocapsid antibodies by sex and age group. B) Weighted seroprevalence of spike antibodies by sex and age group.

We analyzed 4,235 blood samples; median age of participants was 34.0 (SD +27.5) years, and the male-to-female ratio was 0.82 (Appendix). The seroprevalence of N antibodies was 45.3% (95% CI 43.7%–46.8%) and for S antibodies was 95.4% (95% CI 94.7%–96.0%). The age-stratified weighted seroprevalence of N antibodies indicated higher seroprevalences in younger age groups (68.0% [95% CI 64.7%–71.1%] in persons 5–29 years of age) and lower seroprevalences in older age groups (25.7% [95% CI 21.1%–30.7%] in persons >80 years of age) (Figure 1, panel A). Age-stratified weighted seroprevalence of S antibodies was 96.2% (95% CI 95.5%–96.8%) for persons >5 years of age and 71.0% (95% CI 61.5%–79.4%) for persons 0–4 years of age.

**Figure 2:**
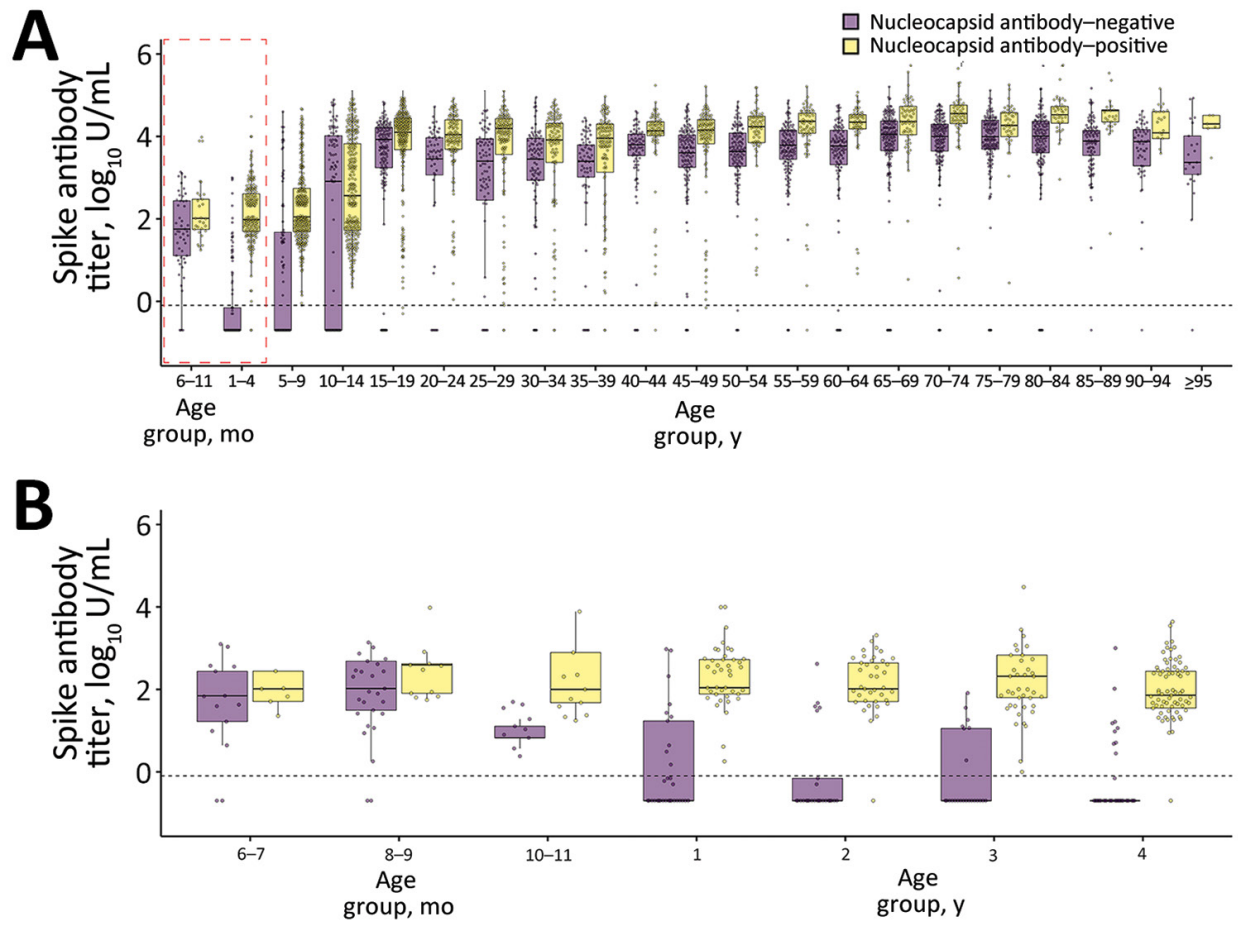
Age-stratified weighted concentrations of spike SARS-Cov-2 antibody stratified by nucleocapsid SARS-Cov-2 antibody seroprevalence in Japan, 2023. A) Spike antibody concentrations stratified by nucleocapsid antibody seroprevalence in persons 6 months to >95 years of age. Red dashed box represents the age group analyzed in panel B. B) Spike antibody concentrations stratified by nucleocapsid antibody seroprevalence in persons 6 months to 4 years of age. Horizonal lines within boxes indicate the median, box tops and bottoms indicate the 75th percentile and 25th percentile, and whiskers extend to 1.5 times the interquartile range (IQR) from 25th percentile and 75th percentile. Dotted horizontal lines represent the cutoff index of 0.8 U/mL. Box plots are weighted with respect to the demographics of Japan.

We measured S antibody concentrations categorized by N antibody seroprevalence status (Figure 2). For persons 6–11 months of age, the estimated weighted median S antibody concentration among N antibody–negative participants was 1.75 U/mL (interquartile range [IQR] 1.14–2.44 U/mL) and among N antibody–positive participants was 2.01 U/mL (IQR 1.77–2.57 U/mL). For persons 1–4 years of age, the estimated weighted median S antibody concentrations among N antibody–negative participants was –0.70 U/mL (IQR -0.70–-0.07 U/mL) and among N antibody–positive participants was 1.98 U/mL (IQR 1.70–2.63 U/mL) (Figure 2, panel A). Overall, for persons >10 years of age, the S antibody concentrations tended to be higher because of immunity from natural infection and vaccination. Antibody concentrations in persons <4 years of age (Figure 2, panel B) suggest the presence of maternally derived immunity; among persons 6–9 months of age, we observed similar S antibody concentrations regardless of N antibody status, whereas we observed a gradual decline in median S antibody concentrations among N antibody-seronegative persons >10 months of age.

## Conclusions

This survey revealed SARS-CoV-2 seroprevalence across various age groups in Japan, with a specific focus on persons 0–15 and >80 years of age, who were not covered in previous surveys. Consistent with prior surveys (surveys 1 and 2), we observed higher N antibody seroprevalence in younger age groups compared with older age groups. Although N antibody seroprevalence is low among older populations, the vaccination coverage among persons >65 years of age during the survey period ranged from 91.5% to 92.8% for the first to third doses, which is higher than for the general vaccinated population (>6 months of age) (68.7%–80.9% for the first to third doses) (*11*). Vaccination coverage for children 5–11 years of age was low; of only 24.1% had first, 23.4% had second, and 9.8% had third doses, and those 6 months–4 years of age had even lower coverage (2.9%–4.0% for the first to third doses) (*11*). Of note, S antibody seroprevalence is higher, especially among persons >10 years of age, because of immunity acquired from infection and vaccination. Therefore, the S antibody seroprevalence in Japan reflects most the younger population acquiring immunity from natural infection and the older population acquiring immunity from vaccination.

Despite low vaccination coverage among children, we observed a high S antibody seroprevalence among children 6–11 months of age at 90.0% (95% CI 55.5%–99.7%). Among the same age group, the N antibody seroprevalence was 40.0% (95% CI 12.2%–73.8%), raising speculation about the presence of maternally derived antibodies in infants. When stratified by N antibody seroprevalence, S antibody concentrations were notably high among N antibody-seronegative persons in the 6–11-month age group (Figure 2, panel B). Although the duration of immunity may vary depending on the mother’s vaccination and infection status (*12*), and a longitudinal study is essential for better understanding, we observed a potential persistence of positive S antibodies until 10–11 months of age, followed by a decline to negative status by 1 year of age.

One limitation of our study is that, despite demographic adjustments using survey weights, biases from the selected clinics in 22 prefectures may not reflect the seroprevalence of the entire population of Japan. To understand the potential effect of selection bias, we are conducting additional surveys simultaneously from different sources. In addition, the proportion does consider the sensitivity or decline of N antibodies (*13*). However, we believe this effect is minimal when using the Roche assay, because only 2.1% of N antibody seroprevalence was reported in December 2021 (*1*) and the assay typically remains positive for ≈2 years (*14*,*15*). Furthermore, the absence of vaccination information for the sampled population hindered a comprehensive understanding of the effect of vaccination on S antibody levels.

Appendix Additional information about infection- and vaccine-induced SARS-CoV-2 seroprevalence, Japan, 2023.
