# Supplementary material for: Infection- and Vaccine-Induced SARS-CoV-2 Seroprevalence, Japan, 2023
Source: Emerg Infect Dis. 2024 Jun;30(6):1267–70. doi: 10.3201/eid3006.231454 (PMC11138984; doi:10.3201/eid3006.231454)
Supplement: Appendix — Additional information about infection- and vaccine-induced SARS-CoV-2 seroprevalence, Japan, 2023. [file 23-1454-Techapp-s1.pdf]

Article DOI: https://doi.org/10.3201/eid3006.231454

EID cannot ensure accessibility for supplementary materials supplied by authors. Readers who have difficulty accessing supplementary content should contact the authors for assistance.

Infection- and Vaccine-Induced SARS-CoV-2 Seroprevalence, Japan, 2023

Appendix

Appendix Table 1. Anti-N antibody seroprevalence

Age	N	Positive	Weighted Seroprevalence (%)*
6–11m	79	29	40.0 (12.2–73.8)
1–4y	306	190	59.8 (49.3–69.6)
5–9y	385	288	74.1 (66.1–81.1)
10–14y	385	284	72.0 (64.4–78.8)
15–19y	385	241	61.1 (53.6–68.3)
20–24y	174	121	67.2 (59.8–74.1)
25–29y	211	139	67.0 (59.6–73.9)
30–34y	196	109	59.6 (52.1–66.7)
35–39y	189	123	65.6 (58.5–72.3)
40–44y	154	78	49.1 (42.3–55.9)
45–49y	231	112	44.9 (39.0–50.9)
50–54y	192	73	40.1 (34.3–46.2)
55–59y	193	68	33.6 (27.6–40.1)
60–64y	162	57	34.0 (27.5–41.0)
65–69y	223	62	28.2 (22.3–34.7)
70–74y	194	56	31.7 (26.3–37.5)
75–79y	191	46	23.9 (18.4–30.3)
80–84y	162	37	23.5 (17.4–30.6)
85–89y	123	24	27.2 (18.9–36.8)
90–94y	73	20	29.2 (17.0–44.1)
95y-	27	6	30.0 (6.7–65.2)

*These values are estimated using weighted tabulation approach and thus the simple proportion does not match.

Appendix Table 2. Anti-N antibody seroprevalence by sex

Age	Sex	N	Positive	Weighted Seroprevalence (%)*
6–11m	Female	36	13	50.0 (6.8–93.2)
6–11m	Male	43	16	40.0 (5.3–85.3)
1–4y	Female	139	90	60.9 (45.4–74.9)
1–4y	Male	167	100	58.8 (44.2–72.4)
5–9y	Female	167	125	72.1 (59.9–82.3)
5–9y	Male	218	163	76.0 (64.7–85.1)
10–14y	Female	178	127	66.2 (54.6–76.6)
10–14y	Male	207	157	77.4 (67.0–85.8)
15–19y	Female	232	139	54.5 (43.6–65.2)
15–19y	Male	153	102	67.4 (56.8–76.8)
20–24y	Female	133	96	73.3 (63.0–82.1)
20–24y	Male	41	25	60.9 (49.9–71.2)
25–29y	Female	130	87	63.3 (52.5–73.2)
25–29y	Male	81	52	70.8 (60.2–79.9)
30–34y	Female	121	68	57.4 (46.8–67.6)
30–34y	Male	75	41	61.1 (50.3–71.2)
35–39y	Female	115	76	65.3 (55.0–74.6)
35–39y	Male	74	47	66.3 (56.1–75.6)
40–44y	Female	91	50	54.9 (45.2–64.2)
40–44y	Male	63	28	43.4 (33.8–53.4)
45–49y	Female	115	52	38.1 (30.0–46.7)
45–49y	Male	116	60	51.4 (42.9–59.8)
50–54y	Female	102	38	42.3 (34.0–50.8)

Age	Sex	N	Positive	Weighted Seroprevalence (%)*
50–54y	Male	90	35	38.2 (29.8–47.1)
55–59y	Female	96	33	31.9 (23.7–41.1)
55–59y	Male	97	35	35.4 (26.6–45.0)
60–64y	Female	88	28	31.8 (23.3–41.4)
60–64y	Male	74	29	37.8 (27.8–48.6)
65–69y	Female	121	35	30.3 (21.8–39.8)
65–69y	Male	102	27	25.9 (18.0–35.2)
70–74y	Female	102	25	24.3 (17.7–32.1)
70–74y	Male	92	31	39.8 (31.5–48.7)
75–79y	Female	116	33	28.0 (20.1–37.0)
75–79y	Male	75	13	20.0 (12.5–29.5)
80–84y	Female	92	18	20.4 (12.9–29.7)
80–84y	Male	70	19	27.8 (17.9–39.6)
85–89y	Female	78	14	26.9 (16.8–39.1)
85–89y	Male	45	10	25.0 (12.1–42.2)
90–94y	Female	54	16	32.4 (17.4–50.5)
90–94y	Male	19	4	21.4 (4.7–50.8)
95y-	Female	22	6	30.0 (6.7–65.2)
95y-	Male	5	0	0.0 (0.0–97.5)

*These values are estimated using weighted tabulation approach and thus the simple proportion does not match.

Appendix Table 3. Anti-S antibody seroprevalence

Age	N	Positive	Weighted Seroprevalence (%)*
6–11m	79	75	90.0 (55.5–99.7)
1–4y	306	219	69.1 (58.9–78.1)
5–9y	385	328	85.3 (78.4–90.7)
10–14y	385	341	87.6 (81.5–92.2)
15–19y	385	367	95.6 (91.4–98.1)
20–24y	174	167	96.0 (92.0–98.4)
25–29y	211	202	95.5 (91.4–98.1)
30–34y	196	188	96.7 (93.0–98.8)
35–39y	189	180	94.9 (90.8–97.5)
40–44y	154	148	95.9 (92.4–98.1)
45–49y	231	226	98.9 (96.9–99.8)
50–54y	192	187	98.2 (95.8–99.4)
55–59y	193	189	98.7 (96.3–99.7)
60–64y	162	154	94.5 (90.4–97.2)
65–69y	223	215	97.7 (94.7–99.2)
70–74y	194	190	98.9 (96.9–99.8)
75–79y	191	185	97.2 (94.0–99.0)
80–84y	162	155	95.9 (91.7–98.3)
85–89y	123	122	100 (96.5–100)
90–94y	73	72	100 (92.6–100)
95y-	27	26	100 (69.2–100)

*These values are estimated using weighted tabulation approach and thus the simple proportion does not match.

Appendix Table 4. Anti-S antibody seroprevalence by sex

Age	Sex	N	Positive	Weighted Seroprevalence*
6–11m	Female	36	35	100 (39.8–100)
6–11m	Male	43	40	100 (47.8–100)
1–4y	Female	139	102	69.6 (54.2–82.3)
1–4y	Male	167	117	68.6 (54.1–80.9)
5–9y	Female	167	143	83.8 (72.9–91.6)
5–9y	Male	218	185	86.7 (76.8–93.4)
10–14y	Female	178	159	87.0 (77.4–93.6)
10–14y	Male	207	182	88.1 (79.2–94.1)
15–19y	Female	232	223	96.6 (90.4–99.3)
15–19y	Male	153	144	94.6 (87.8–98.2)
20–24y	Female	133	128	95.6 (89.0–98.8)
20–24y	Male	41	39	96.6 (90.3–99.3)
25–29y	Female	130	124	94.4 (87.5–98.2)
25–29y	Male	81	78	96.6 (90.5–99.3)
30–34y	Female	121	118	98.9 (94.2–100)
30–34y	Male	75	70	93.3 (86.1–97.5)

Age	Sex	N	Positive	Weighted Seroprevalence*
35–39y	Female	115	111	95.9 (89.9–98.9)
35–39y	Male	74	69	91.8 (84.5–96.4)
40–44y	Female	91	89	99.1 (95.2–100)
40–44y	Male	63	59	94.3 (88.1–97.9)
45–49y	Female	115	111	98.6 (94.9–99.8)
45–49y	Male	116	115	99.3 (96.2–100)
50–54y	Female	102	98	97.9 (94.0–99.6)
50–54y	Male	90	89	99.2 (95.8–100)
55–59y	Female	96	95	99.2 (95.4–100)
55–59y	Male	97	94	98.2 (93.8–99.8)
60–64y	Female	88	83	94.5 (88.5–98.0)
60–64y	Male	74	71	93.3 (86.1–97.5)
65–69y	Female	121	117	97.2 (92.2–99.4)
65–69y	Male	102	98	97.2 (92.1–99.4)
70–74y	Female	102	99	98.6 (95.2–99.8)
70–74y	Male	92	91	99.2 (95.9–100)
75–79y	Female	116	111	95.8 (90.4–98.6)
75–79y	Male	75	74	98.9 (94.3–100)
80–84y	Female	92	91	99.0 (94.4–100)
80–84y	Male	70	64	93.1 (84.5–97.7)
85–89y	Female	78	77	100 (94.6–100)
85–89y	Male	45	45	100 (90.3–100)
90–94y	Female	54	53	100 (89.7–100)
90–94y	Male	19	19	100 (76.8–100)
95y-	Female	22	21	90 (55.5–99.7)
95y-	Male	5	5	100 (2.5–100)

*These values are estimated using weighted tabulation approach and thus the simple proportion does not match.

Appendix Table 5. Anti-S antibody titer among anti-N antibody negative

Age	Q1	Median	Q3	IQR
6–11m	1.14	1.75	2.44	1.30
1–4y	−0.70	−0.70	−0.07	0.63
5–9y	−0.70	−0.70	1.67	2.37
10–14y	−0.70	2.95	4.02	4.72
15–19y	3.28	3.93	4.22	0.93
20–24y	3.07	3.45	3.98	0.91
25–29y	2.58	3.45	3.98	1.40
30–34y	2.95	3.45	3.95	1.00
35–39y	3.02	3.40	3.83	0.81
40–44y	3.54	3.81	4.06	0.52
45–49y	3.26	3.61	4.04	0.78
50–54y	3.29	3.65	4.08	0.79
55–59y	3.46	3.79	4.15	0.69
60–64y	3.35	3.77	4.15	0.80
65–69y	3.66	4.05	4.38	0.72
70–74y	3.67	4.00	4.30	0.63
75–79y	3.71	4.00	4.39	0.68
80–84y	3.60	4.05	4.37	0.76
85–89y	3.54	3.89	4.14	0.60
90–94y	3.48	3.88	4.16	0.68
95y-	3.08	3.37	4.01	0.93

Appendix Table 6. Anti-S antibody titer among anti-N antibody positive

Age	Q1	Median	Q3	IQR
6–11m	1.77	2.01	2.57	0.81
1–4y	1.70	1.98	2.63	0.93
5–9y	1.69	2.05	2.74	1.05
10–14y	1.74	2.57	3.82	2.08
15–19y	3.68	4.12	4.45	0.77
20–24y	3.69	4.05	4.40	0.71
25–29y	3.57	4.19	4.43	0.87
30–34y	3.37	3.91	4.32	0.95
35–39y	3.18	3.96	4.30	1.12
40–44y	4.02	4.14	4.35	0.33

Age	Q1	Median	Q3	IQR
45–49y	3.83	4.15	4.41	0.58
50–54y	3.85	4.24	4.49	0.64
55–59y	4.08	4.37	4.57	0.49
60–64y	4.19	4.37	4.53	0.34
65–69y	4.04	4.36	4.72	0.68
70–74y	4.34	4.56	4.76	0.42
75–79y	4.00	4.27	4.58	0.57
80–84y	4.35	4.53	4.73	0.37
85–89y	4.39	4.63	4.65	0.26
90–94y	3.95	4.22	4.61	0.66
95y-	4.22	4.41	4.51	0.29

Appendix Table 7. Anti-S antibody titer among anti-N antibody positive (≤4 y old)

Age	Q1	Median	Q3	IQR
6–7m	1.92	2.01	2.23	0.31
8–9m	2.26	2.59	2.62	0.37
10–11m	1.72	2.31	2.90	1.18
1y	1.95	2.05	2.72	0.77
2y	1.71	2.01	2.67	0.96
3y	1.84	2.32	2.89	1.06

Appendix Table 8. Anti-S antibody titer among anti-N antibody positive (≤4 y old)

Age	Q1	Median	Q3	IQR
6–7m	1.65	2.14	2.44	0.78
8–9m	1.55	2.16	2.67	1.12
10–11m	0.83	0.83	1.11	0.28
1y	−0.70	−0.70	1.26	1.96
2y	−0.70	−0.70	−0.15	0.54
3y	−0.70	−0.70	1.08	1.78
